# Combination of traditional Chinese bone setting and NMES technique for treating lumbar disc herniation: a case report

**DOI:** 10.3389/fresc.2024.1465623

**Published:** 2024-12-05

**Authors:** Yimei Bao, ZhiJin Wang

**Affiliations:** ^1^ Rehabilitation Medicine Department, Dalian Fourth People’ Hospital, Dalian, Liaoning, China; ^2^ Department of Spine Surgery, Central Hospital of Dalian University of Technology, Dalian, Liaoning, China

**Keywords:** lumbar disc herniation, NMES, manual therapy, spinal internal balance disorder, low back pain

## Abstract

**Objective:**

To analyze and study the causes and treatment approaches for lumbar disc herniation, focusing on office workers.

**Methods:**

The concept of spinal internal balance disorder as a foundation for treating traumatic spinal diseases was introduced. Pathological changes occurring with single (or multiple) vertebral displacement were considered. For the first time, the “spinal (point) rotation repositioning method” from traditional Chinese bone setting was combined with NMES (neuromuscular electrical stimulation) technology to treat low back pain.

**Results:**

The patient's symptoms of low back pain were cured within a short period, allowing a quick return to work and daily life, with no recurrence of the disease observed during long-term follow-up.

**Conclusion:**

The combination of traditional Chinese bone setting and NMES provides a more effective and quicker resolution to low back pain in office workers with lumbar disc herniation. This integrated approach not only relieves pain but also restores lumbar function, ensuring long-term stability and reducing recurrence**.**

## Introduction

The incidence of low back pain has significantly increased, rising by 18% from 2006 to 2016, attributable to changes in living and working environments as well as the accelerated pace of modern society. Low back pain is classified internationally based on its duration into three categories: acute (lasting less than 4 weeks), subacute (4–12 weeks), and chronic (exceeding 12 weeks) (1). It is characterized as pain located below the ribcage, encompassing the lumbosacral and sacroiliac regions, and may be accompanied by radiating pain extending into the lower limbs. This condition is a prevalent health concern among adults, with an incidence rate reported as high as around 80% (2, 3). Low back pain not only adversely affects patients' health, quality of life, and work performance but also imposes a substantial economic burden on society, manifested through increased medical costs and indirect social expenses**.**

In the United States, the lifetime prevalence of low back pain is approximately 90%, with an annual prevalence rate of 50% (2). In China, the predominant causes of low back pain include chronic strain, spinal degeneration, bone spurs, and intervertebral disc herniation. Additionally, a smaller subset of patients experiences low back pain as a result of trauma, fractures, tumors, or infections such as tuberculosis (4). The sedentary nature of modern work environments has led to sitting becoming the most common posture among office workers. A considerable proportion of individuals spend over 8 h daily in a seated position, with reported rates of low back pain among office workers ranging from 25% to 51% (5).

The pathogenesis of degenerative spine disease is multifactorial, influenced by age, genetic predisposition, poor posture, and lifestyle choices (6). This condition typically presents as disc degeneration, osteoarthritis, and spinal instability, which can culminate in nerve root compression and chronic pain (7). As societal lifestyles continue to evolve, degenerative spine disease has exerted a significant impact on healthcare systems, resulting in escalated medical expenditures and heightened demand for healthcare services (8).

Historically, the understanding of low back pain can be traced back over 2,000 years. The “Fifty-Two Diseases Prescription,” dating to the 2nd century BC, documented conditions associated with cervical spondylosis and low back pain. In 1929, Dandy reported the first surgical intervention for lumbar disc pathology, initially misdiagnosed as a spinal tumor or chondroma. Subsequently, in 1934, Mixter and Barr established a morphological correlation between disc tissue and lower back pain, leading to the foundational concept of “lumbar disc herniation.” This concept encompasses the pathological diagnosis whereby herniation of the lumbar disc's nucleus pulposus compresses the sciatic nerve root, resulting in nerve dysfunction or pain (9, 10).

## Case report

### Patient information

The patient is a 33-year-old female with no significant prior medical or pharmacological history. Currently employed in a white-collar position, she spends extensive hours seated at a computer desk. Approximately three months ago, she began experiencing low back pain attributed to prolonged sitting, which worsened after lifting a heavy object just one day prior to her visit. She now reports restricted forward bending, accompanied by radiating pain in the lower limbs. The Japanese Orthopaedic Association (JOA) lumbar score was recorded at 19 points. The patient had previously received various treatments, including medication, massage, and physical therapy; however, these provided only partial relief, and her symptoms fluctuated. In pursuit of a more definitive diagnosis and effective treatment, she presented to our hospital**.**

### Physical examination

There was tenderness on the left side of the lumbar spinous processes, with no abnormalities in skin sensation or muscle strength in both lower limbs. The straight leg raising test was positive.

### Examination results

**Figure 1:**
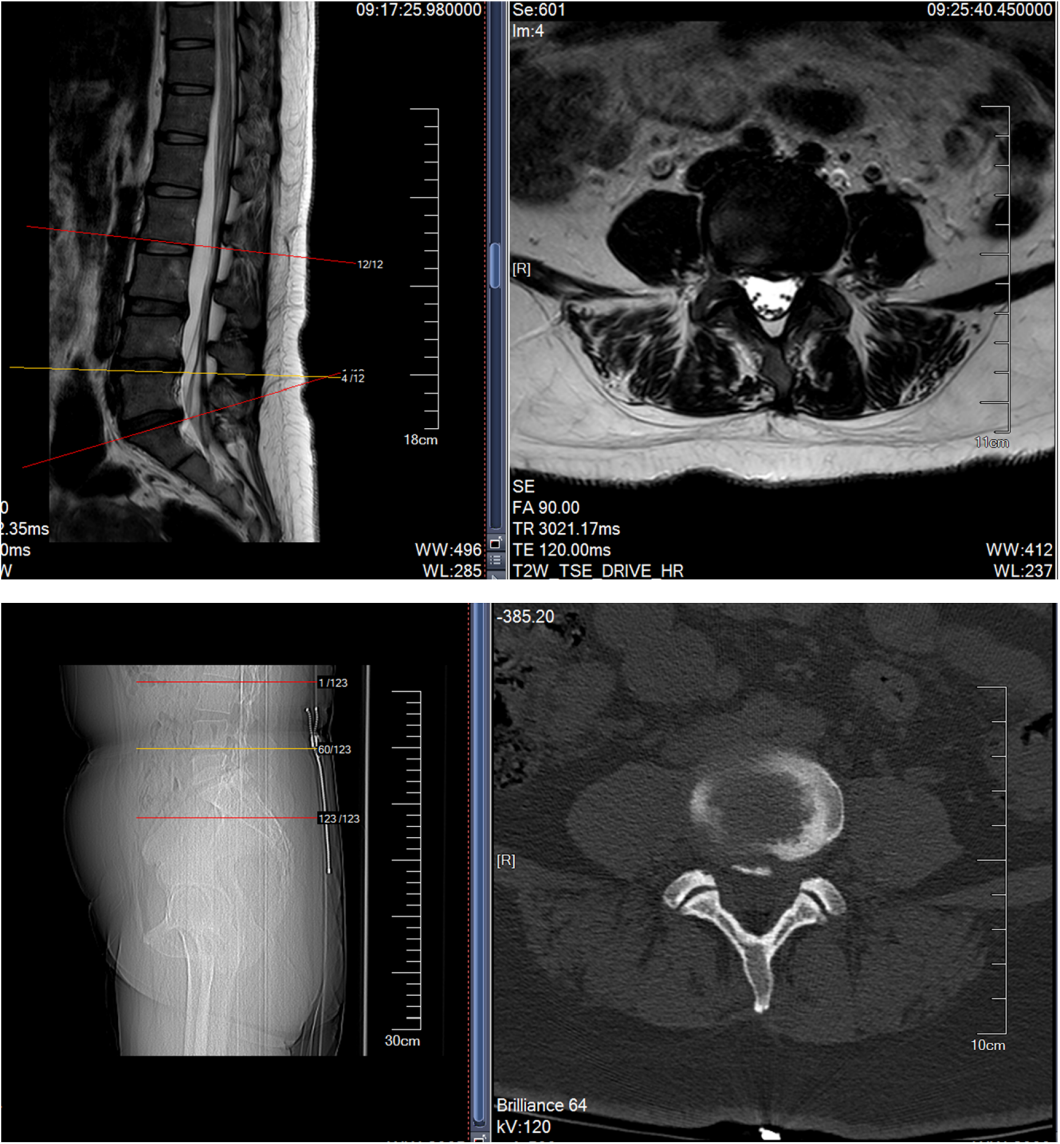
Shows that MRI indicates herniation of the L3/4, L4/5 and L5/S1 intervertebral discs, while CT suggests calcification of the posterior ligament.

Lumbar CT and MRI showed lumbar disc protrusion at L3/4, L4/5, and L5/S1, compressing the dural sac (see MRI and CT images below Figure 1).

### Clinical diagnosis

Lumbar disc herniation (L3/4, L4/5, L5/S1).

### Patient's request

The patient, a 33-year-old office worker, expressed significant distress over how her persistent low back pain was disrupting both her personal life and professional responsibilities. Despite trying various conventional treatments such as medication, massage, and physical therapy, she only experienced temporary relief. Her primary concerns were the recurring nature of the pain and its growing interference with her ability to carry out daily activities without discomfort. As a result, she actively sought a more effective and long-lasting treatment that would enable her to return to normal life, including her desk job, without the fear of further aggravating her condition. After starting the combined treatment of Traditional Chinese Bone Setting and NMES, she felt hopeful due to the rapid improvement in her mobility and a reduction in pain levels.

### Initial assessment

Considering the diagnosis of lumbar disc herniation, the hospital recommended bed rest, lumbar support, and oral medication for promoting blood circulation and removing stasis, but the condition fluctuated.

### Re-assessment

**Figure 2:**
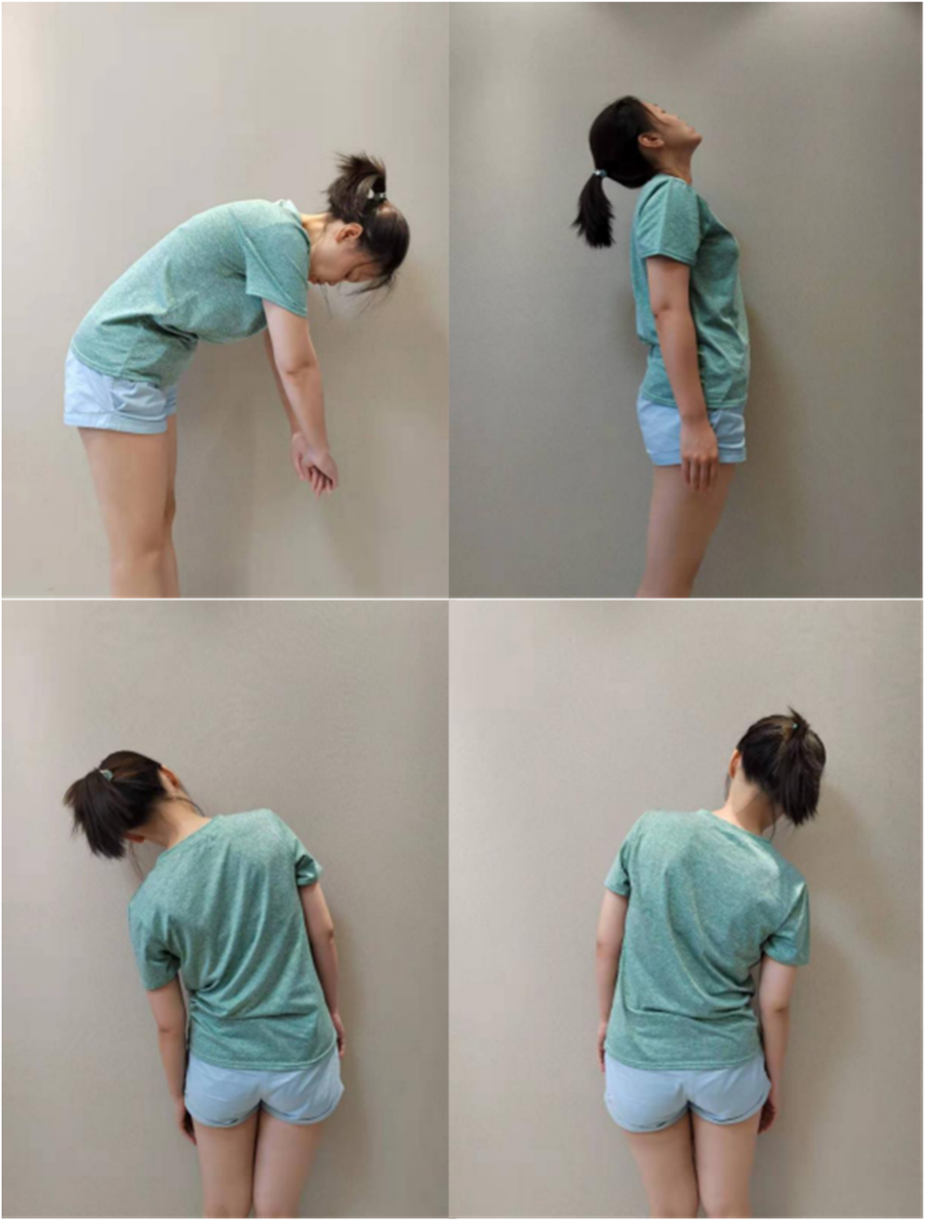
Assessment of Patient's posture and function before treatment.

The patient, a white-collar worker characterized by prolonged periods of sitting and a lack of regular exercise, underwent a comprehensive re-evaluation. The clinical diagnosis was established as follows: (1) Lumbar muscle strain; (2) Lumbar disc herniation at the levels of L3/4, L4/5, and L5/S1. Upon analysis, it was observed that the patient's deep lumbar muscles exhibited stiffness with diminished elasticity, resulting in a reduced capacity to stabilize the spinal joints. Cumulative strain and/or degenerative changes appeared to compromise spinal stability. Additionally, the act of lifting heavy objects without appropriate adjustments to both internal and external postural factors likely contributed to localized soft tissue damage. The patient reported a visual analog scale (VAS) score of 6 for low back pain, accompanied by left-sided lumbar muscle spasms and varying degrees of restriction in flexion, extension, lateral bending, and rotation (refer to the Figure 2).

### Potential complications

While the treatment plan demonstrates a high success rate, potential complications warrant careful monitoring. These may include localized skin discomfort or allergic reactions resulting from electrical stimulation, temporary muscle soreness or stiffness following manual therapy, and, in rare instances, nerve damage or further spinal misalignment due to improper treatment techniques. It is crucial to closely monitor the patient's response to therapy, allowing for timely adjustments to the intensity and frequency of treatment as necessary.

### Follow-up strategy

As the treatment progresses, regular follow-up appointments will be scheduled to assess the efficacy of the therapeutic interventions and to make necessary adjustments to the treatment plan. The follow-up strategy will involve a gradual reduction in the frequency of neuromuscular electrical stimulation (NMES) sessions, along with the incorporation of rehabilitation exercises designed to strengthen the lower back musculature. Furthermore, the patient will receive guidance on modifying her work habits to mitigate prolonged sitting and to prevent the recurrence of symptoms. In the long term, ongoing imaging studies will be conducted to evaluate improvements in the condition of the intervertebral discs, which will inform subsequent treatment strategies. This personalized and integrative treatment approach is aimed at enhancing the patient's prognosis while minimizing the risk of recurrence associated with lumbar disc herniation. The comparison of VAS scores and functional activity scores of the patient before and after treatment can be found in Table 1.

**Table 1:** Illustrates the comparison of the patient's VAS scores and functional activity before and after treatment.

Lumbar VAS and mobility score table					
	VAS Score (points)	Flexion (0–90°)	Extension (0–30°)	Lateral Bending (0–30°)	Rotation (0–30°)
		Left	Right	Left	Right
Initial	6	45	10	25	15
Follow-up	0	80	30	25	25

### Clinical hypothesis

The strength of the paraspinal muscles is crucial for maintaining lumbar stability (6). Research indicates that prolonged sitting and poor hip flexion postures can lead to the shortening of posture-maintaining muscles, such as the iliopsoas (7). Poor posture contributes to improper muscle contraction patterns, which increases the risk of muscle strength reduction and imbalance. The shortening of lumbar and abdominal muscles can result in lumbar lordosis and pelvic tilt, leading to compensatory weakness in the glutes, erector spinae, and multifidus muscles that are responsible for maintaining posterior pelvic tilt and spinal extension. Such poor postural habits or excessive loads can easily cause injury to these muscles, thereby resulting in low back pain.

**Figure 3:**
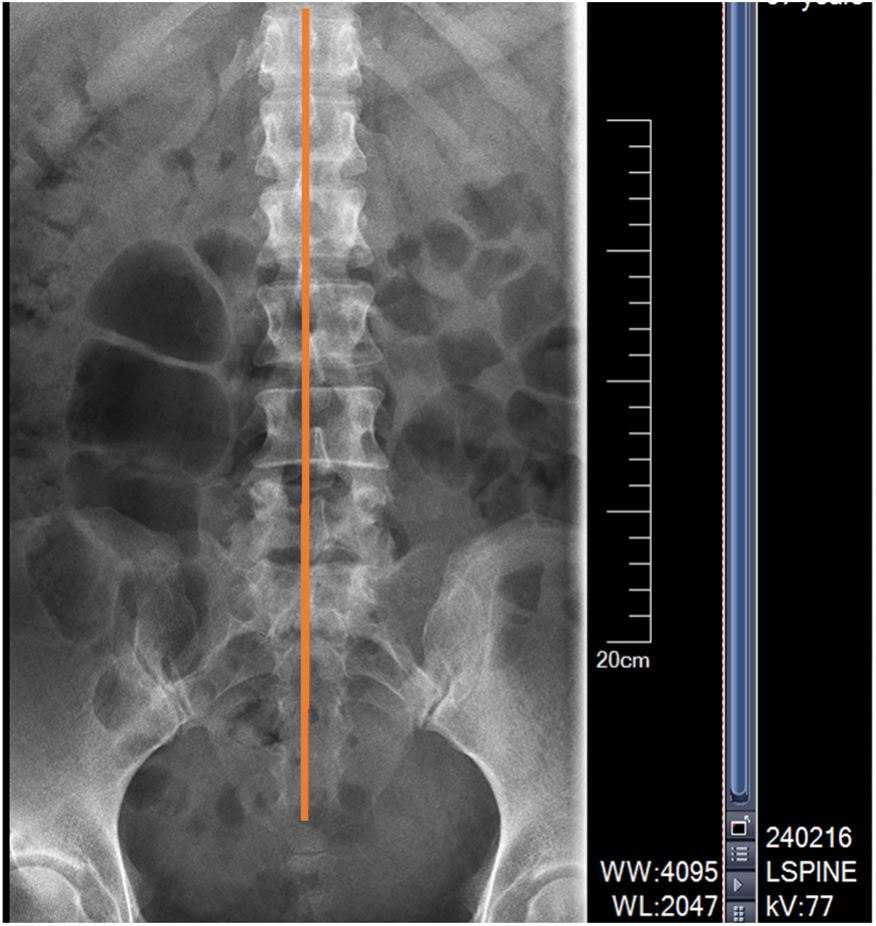
X-ray anteroposterior view indicates: spinous process is not centered, showing signs of rotation.

**Figure 4:**
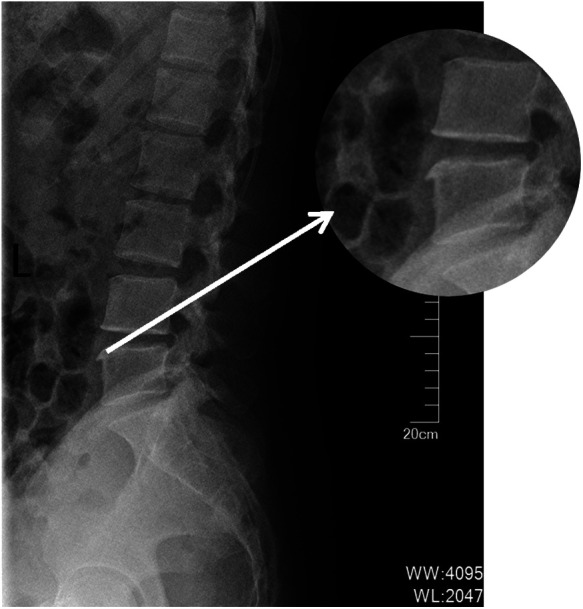
Lateral view indicates: osteophyte formation on the vertebral body, enhancing vertebral stability.

Extended periods of sitting contribute to muscle imbalances, which may cause single or multiple vertebral displacements (see Figures 3, 4 below), facet joint misalignments, and alterations in ligament and disc tension. These changes can reduce the size of the intervertebral foramen and spinal canal, disrupting the internal balance of the spine and causing disturbances in the external balance (muscle scaffold) (9). Conventional treatments for low back pain often focus on muscle-targeted interventions, such as massage, acupuncture, and blood-activating medications, or surgical options for disc issues. However, treatments aimed at correcting both spinal internal and external balance are rarely employed, which can lead to unsatisfactory outcomes or recurrence of the condition.

In light of this, we propose a clinical hypothesis: First, relax the muscles to alleviate spasms and address imbalances; second, utilize traditional Chinese bone setting techniques to correct any single or multiple vertebral displacements; and finally, implement a strengthening regimen for the muscles to achieve and maintain both spinal internal and external scaffold balance. This comprehensive approach is designed to ensure recovery from low back pain without recurrence.

## Treatment plan

### Treatment methods

1. Muscle Relaxation: Employ neuromuscular electrical stimulation (NMES) technology to alleviate local muscle spasms, effectively reducing muscle tone and promoting relaxation.
2. Manual Therapy: Implement spinal rotation repositioning techniques. During this process, the clinician shifts the patient's center of gravity onto the affected vertebral facet joint, temporarily destabilizing the spine. Subsequently, the clinician manually corrects the displaced spinous process. The application of direct traction and rotational forces by the clinician's thumb facilitates easier repositioning of the affected vertebra, utilizing mechanical forces. This technique is characterized by its steadiness, precision, and skillfulness, resulting in the correction of vertebral displacement and immediate relief from low back and leg pain.
3. Functional Exercises: Instruct the patient to utilize NMES for abdominal strengthening, perform back extensions on a yoga ball, engage in dynamic yoga ball planks, and participate in other core muscle training exercises.
4. Health Education: Encourage the patient to return to work promptly while avoiding prolonged periods of standing or sitting, heavy lifting, and twisting motions of the waist. Recommend frequent changes in posture during office work, taking brief breaks to move around, utilizing chairs with adequate back support, and regularly adjusting the office chair for optimal ergonomics. Provide guidance on proper lifting techniques, emphasizing hip and knee flexion while maintaining a straight back and engaging the abdominal muscles. Suggest the use of a medium-firm mattress to enhance pain relief. Promote regular exercise targeting the trunk, gluteal muscles, and abdominal muscles, including activities such as walking, swimming, and back extension exercises.

### NMES application

**Figure 5:**
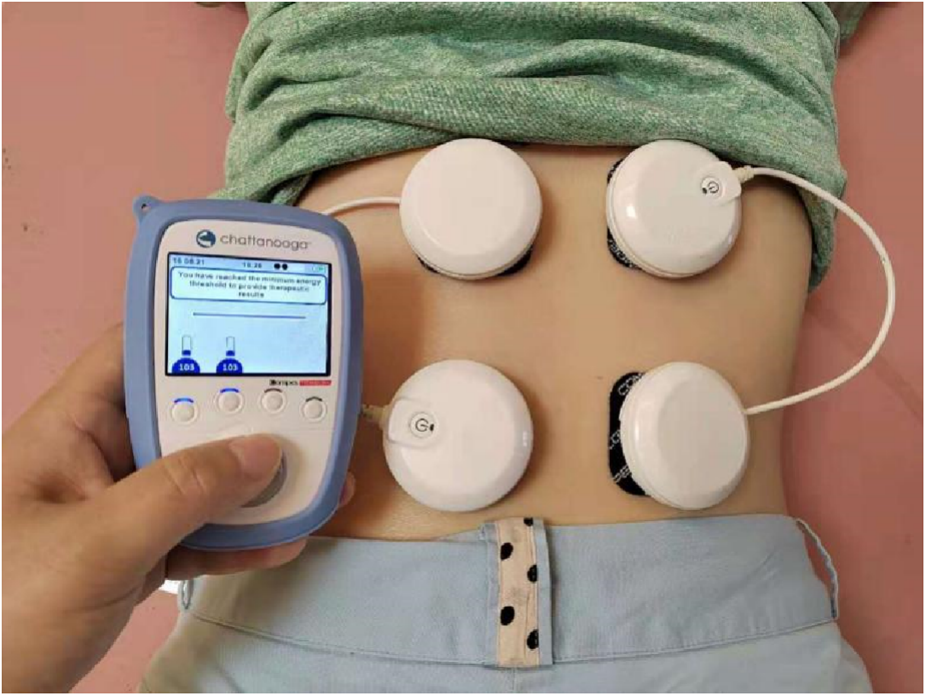
Shows the placement of the NMES device.

We employed neuromuscular electrical stimulation (NMES) technology in decontraction mode to effectively reduce muscle spasms in the erector spinae. This approach facilitates the release of endorphins, thereby alleviating both pain and anxiety. Additionally, we incorporated lumbar muscle stretching exercises to enhance blood circulation in the affected area. The NMES sessions can be administered 1–2 times daily, with each session lasting approximately 20 min. Electrodes are strategically placed on both sides of the erector spinae muscle belly to maximize therapeutic efficacy. The operation demonstration image is shown in Figure 5 below.

### Electrode placement

Electrodes were placed on both sides of the erector spinae muscle belly.

### Post-Manual therapy

**Figure 6:**
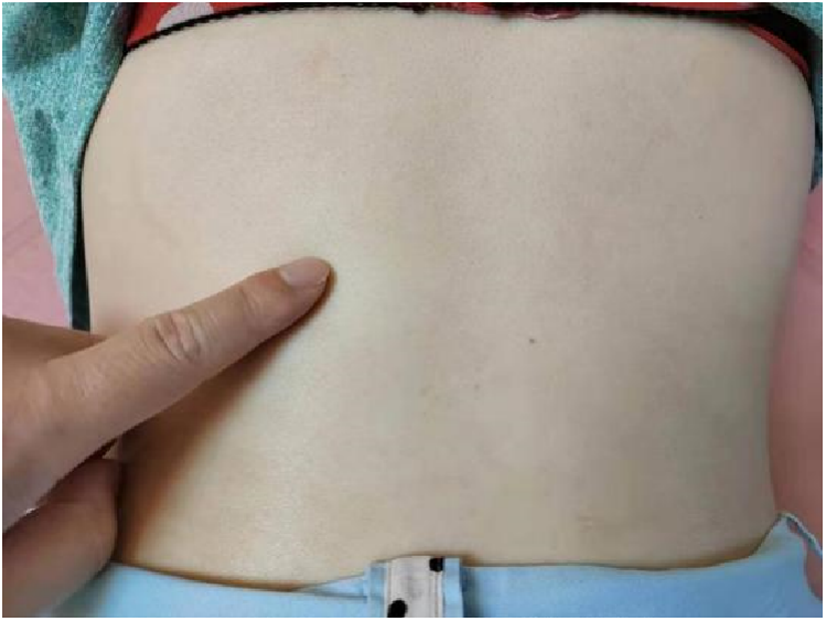
Left lumbar muscle spasm.

**Figure 7:**
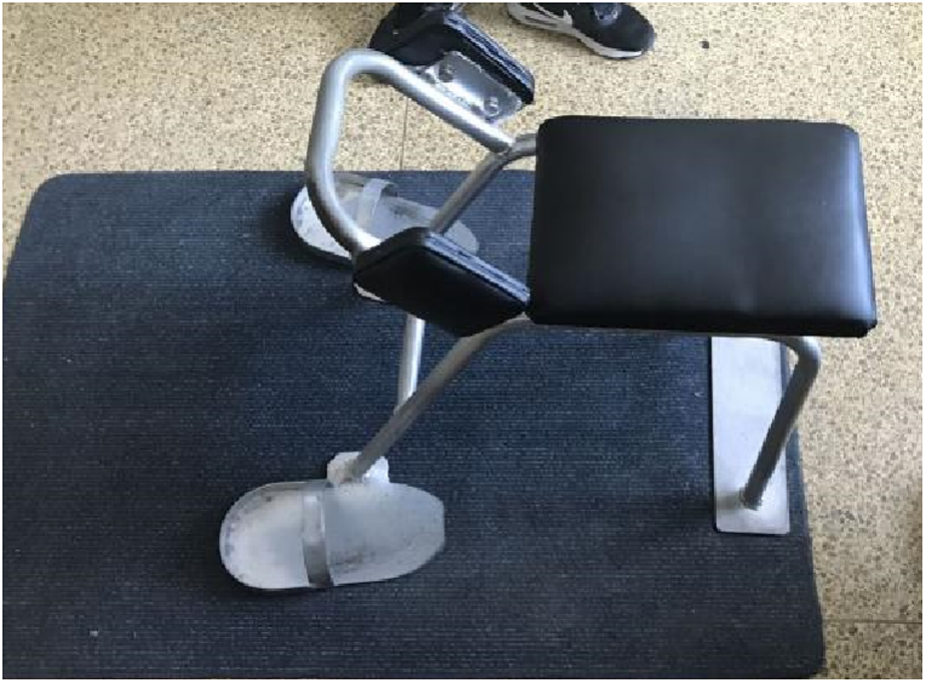
New orthopedic manipulation uses an orthopedic chair.

After the manual therapy successfully corrected vertebral displacement, NMES lumbar stabilization programs were implemented to enhance core muscle strength. (Figures 6, 7 shows the patient's lumbar muscle spasm and the orthopedic bench used in the osteopathy treatment.) The treatment regimen consisted of 3–5 sessions per week over a duration of 4 weeks. The exercises incorporated into the program included standard lumbar and abdominal strengthening routines, which were synchronized with electrical stimulation during the contraction phase to facilitate eccentric resistance training. This was subsequently followed by a relaxing massage program aimed at promoting muscle relaxation and recovery.

### Results

**Figure 8:**
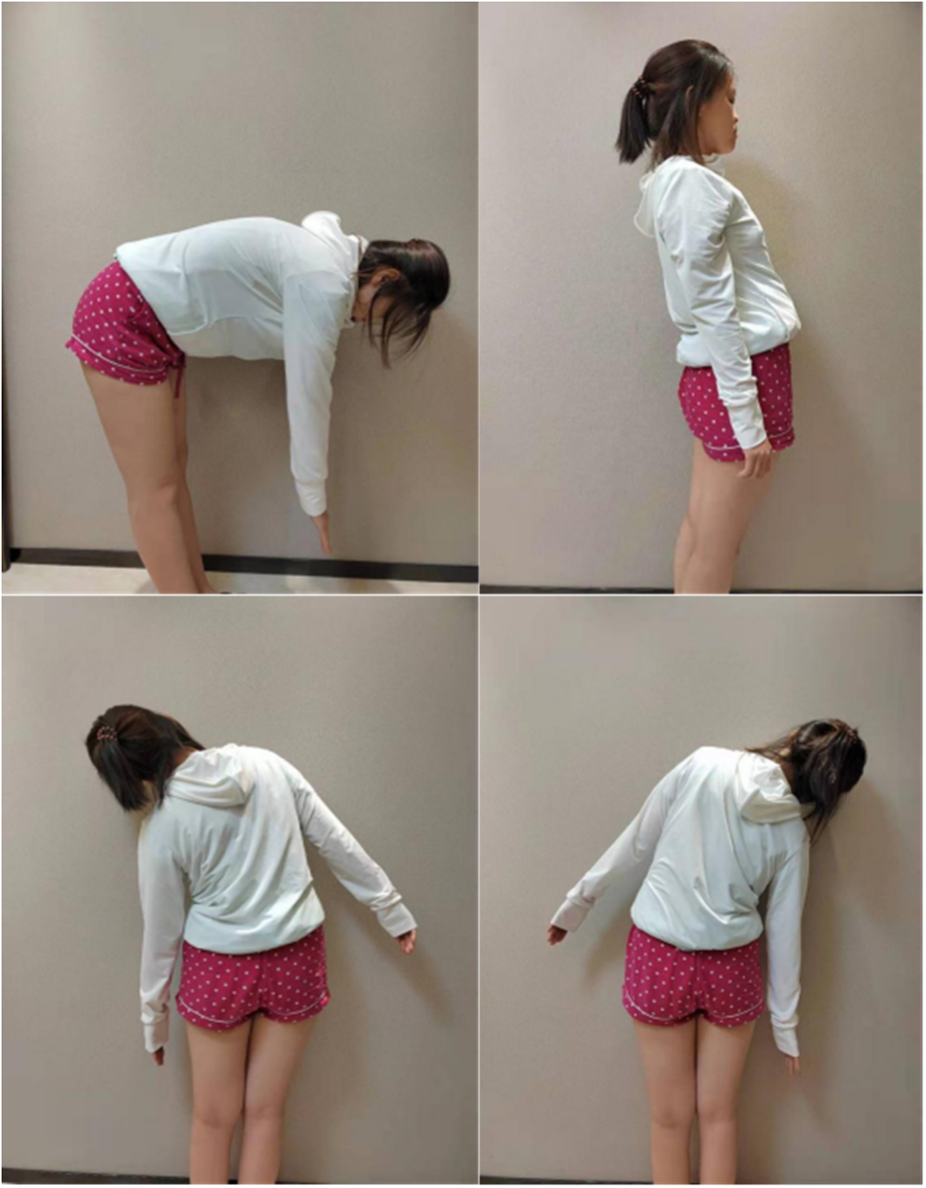
Post-Treatment functional assessment of the patient.

After three treatments, the patient's lumbar region showed no tenderness, muscle spasms were relieved, and normal flexion, extension, and lateral bending were restored. The patient's motor function significantly improved after treatment, as shown in Figure 8.

The patient was satisfied with the treatment results and reported no recurrence of low back pain during follow-up a year later, with a quick return to daily life and work (Table 2 summarizes the patient's treatment results).

**Table 2:** Comparison of data before and after treatment for lumbar disc herniation.

Parameter	Before treatment	After treatment	Comments
VAS Score	6	0	Complete pain relief achieved.
Flexion (degrees)	45	80	Significant improvement in forward bending.
Extension (degrees)	10	30	Enhanced backward bending capability.
Lateral Bending (degrees)	Left: 25, Right: 15	Left: 25, Right: 25	No change in left lateral bending; right improved.
Patient Satisfaction	Low	High	Patient reported satisfaction with treatment results.
Recurrence of Symptoms	Yes	No	No recurrence during one-year follow-up.
Return to Work	Delayed	Quick return	Patient was able to resume normal activities promptly.

## Discussion

The annual incidence rate of low back pain is increasing, with a younger demographic increasingly affected. Many office workers with prolonged sitting suffer from low back pain, often diagnosed as lumbar disc herniation or muscle strain. Traditional treatments like dehydration, steroids, and bed rest sometimes fail to achieve significant clinical results.

The Definition of “Lumbar Disc Herniation” in New Medical Orthopedics: The discipline of New Medical Orthopedics originated in the late 1960s, during the early stages of integrated Chinese and Western medicine in China. Professor Feng Tianyou studied and researched traditional Chinese folk medicine while incorporating modern medical knowledge of anatomy, physiology, pathology, and biomechanics. He discovered that vertebral misalignment does exist but is not as simplistically described in traditional Chinese medicine as “tendons slipping out of place” or “bones out of alignment.” Instead, he proposed the theory of “vertebral displacement,” introducing a new diagnostic standard for identifying and assessing whether and how vertebrae are displaced. The intervertebral discs and facet joints form the basis for spinal movement. The intervertebral disc, which acts as an elastic cushion, consists of cartilage plates, the annulus fibrosus, and the nucleus pulposus. The nucleus pulposus, facet joints, and surrounding ligaments are closely connected and maintain stability in the intervertebral joints in a balanced manner, regardless of the position of the spine. This forms the internal balance of the spine. The muscles in the front, back, and sides of the spine help maintain its coordination and stability in various positions, which is known as the spine's external balance. The coordinated harmony between the spine's internal and external balance is crucial for the body's functional activities.

When the vertebrae, intervertebral discs, intervertebral ligaments, and other structures that make up the spine experience cumulative strain and/or degeneration (particularly in the intervertebral discs), the stability of the spine may weaken. If subjected to external trauma or sudden twisting forces, it can result in slight displacement of one or more vertebrae. This displacement alters the tension in the facet joints, intervertebral ligaments, and intervertebral discs, disrupting the internal balance of the spine and triggering corresponding changes in the external balance (the muscular framework).

In response to alleviate and/or eliminate pressure on nerves or blood vessels, the body may temporarily develop compensatory mechanisms, such as muscle stiffness or changes in spinal curvature. Over time, compensatory changes like bone spurs, ligament calcification, and disc herniation may occur. When these compensatory mechanisms are effective, neurological dysfunction may not manifest. However, once compensation fails, it results in irritation or compression of surrounding tissues, leading to corresponding symptoms. This concept of “lumbar disc herniation” was introduced by Professor Feng Tianyou (11).

Professor Tianyou Feng's theory of spinal internal and external balance disorder provides a theoretical basis for traumatic spinal diseases. Single (or multiple) vertebral displacement is the main pathological change. Using the “spinal (point) rotation repositioning method” has shown good clinical results in treating muscle strain, lumbar disc herniation, cervical spondylosis, and atlantoaxial subluxation (12).

Accumulative strain (repeated bending, prolonged sitting) or degeneration (aging) weakens spinal stability. In specific postures (like bending), internal balance (spine) lacks the protection of the external balance scaffold (core muscle strength), or sudden posture changes disrupt spinal internal and external balance, causing focal soft tissue damage and vertebral displacement. Joint facet misalignment and changes in ligament and disc tension reduce the intervertebral foramen and spinal canal size, resulting in lumbar muscle stiffness, pain, limited movement, and compensatory spinal curvature.

NMES is a CFDA-approved method for muscle relaxation and training, applying electrical stimulation to trigger points (using electrodes placed on the skin) to induce muscle contraction either directly or through neural control, commonly used to treat increased muscle tone and disuse muscle atrophy, maintaining normal muscle function (13).

Based on these principles, a treatment plan was developed. NMES technology was first used to relieve paraspinal muscle spasms (like the erector spinae), preparing for manual correction through point-by-point spinal rotation. NMES was then used to strengthen paraspinal muscles, achieving joint stability. Advantages of NMES training include: (1) Early effective relief of muscle spasms in strained lumbar muscles. (2) Early isometric contraction training for stabilizing muscles, reducing mechanical stress on spinal joints. (3) Lumbar muscles primarily composed of type I high-endurance muscle fibers (14), with NMES programs targeting type I muscle fiber training for efficient workouts. (4) NMES training promotes motor relearning and posture control through synchronized electrical stimulation and voluntary muscle contraction.

The advantages of NMES technology include reduced muscle tone, enhanced muscle strength, and pain management. Different modes are selected based on the patient's condition. As patients are mostly office workers with limited treatment time, they can continue using NMES at home as advised, potentially achieving a complete cure for low back pain.

## Data Availability

The original contributions presented in the study are included in the article/Supplementary Material, further inquiries can be directed to the corresponding author.
